# Author Correction: Revisiting the pH-gated conformational switch on the activities of HisKA-family histidine kinases

**DOI:** 10.1038/s41467-020-16292-8

**Published:** 2020-05-19

**Authors:** Cristina Mideros-Mora, Laura Miguel-Romero, Alonso Felipe-Ruiz, Patricia Casino, Alberto Marina

**Affiliations:** 1 Instituto de Biomedicina de Valencia, Consejo Superior de Investigaciones Científicas (IBV-CSIC), Jaume Roig 11, 46010 Valencia, Spain; 2 Universidad UTE, Facultad de Ciencias de la Salud Eugenio Espejo, Rumipamba s/n, Quito, Ecuador; 3 Departament de Bioquímica i Biología molecular, Universitat de València, Dr. Moliner 50, 46100 Burjassot, Spain; 4 Estructura de Recerca Interdisciplinar en Biotecnologia i Biomedicina (ERI BIOTECMED), Universitat de València, Dr Moliner 50, 46100 Burjassot, Spain; 5 CIBER de enfermedades raras (CIBERER-ISCIII), Madrid, Spain; 6 Present Address: Institute of Infection, Inmmunity and Inflammation, University of Glasgow, Glasgow, G12 8TA UK

**Keywords:** Biochemistry, X-ray crystallography, Microbiology

Correction to: *Nature Communications* 10.1038/s41467-020-14540-5, published online 7 February 2020.

The original version of this Article omitted a reference to previous work in ‘Chakraborty, S., Winardhi, R.S., Morgan, L.K., Yan, J. & Kenney, L.J. Non-canonical activation of OmpR drives acid and osmotic stress responses in single bacterial cells. *Nat. Commun*. **8**, 1587 (2017)’. This has been added as reference 29 after the fourth sentence in the Results paragraph ‘pH Influence on the functional activities of EnvZ-OmpR’: ‘A substantial reduction in EnvZ autophosphorylation induced by lowering the pH (from 7.5 to 5.5) has also been demonstrated previously^29^.’ This has been corrected in the PDF and HTML versions of the Article.

