# Supplementary material for: Revisiting the pH-gated conformational switch on the activities of HisKA-family histidine kinases
Source: Nat Commun. 2020 Feb 7;11:769. doi: 10.1038/s41467-020-14540-5 (PMC7005713; doi:10.1038/s41467-020-14540-5)
Supplement: Supplementary file 1 — Supplementary Information [file 41467_2020_14540_MOESM1_ESM.pdf]

Supplementary Information

Revisiting the pH-gated conformational switch on the activities of HisKA-family histidine kinases

Cristina Mideros-Mora et al.

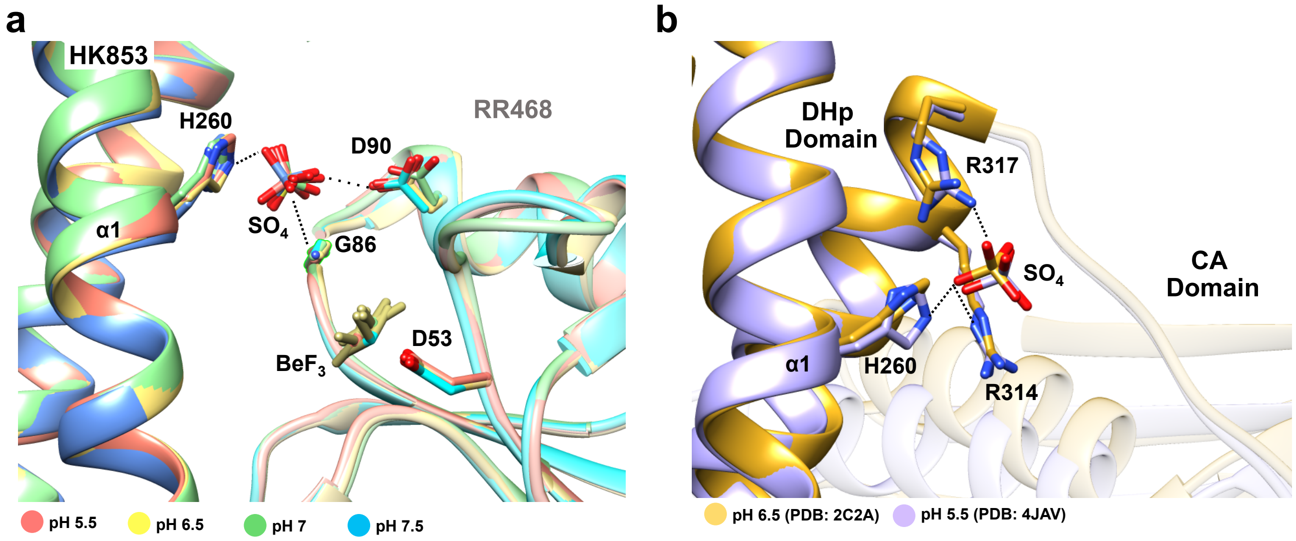

Supplementary Figure 1. Stabilization of sulfate ion bound to the phosphorylatable His260 in HK853. **a** Close view of the active center of superposed structures HK853-RR468 complexes solved at different pHs (colour code is shown at the bottom) shows that the sulfate ion interacting with H260 in *gauche-* conformation is stabilized by additional interactions with residues G86 and D90 of RR468. Contacts are denoted by dotted lines. Structures are shown in cartoon with the interacting residues and the sulfate ions with carbons in the same color as the corresponding molecule. The phosphomimetic BeF_3^- found at the active center of RR468 molecules are shown in sticks as well as the phosphorylatable RR468 D53. **b** Close view of HK853 structures where a sulfate ion is found interacting with the H260 (PDBs 2C2C and 4JAV in orange and purple, respectively). In these structures H260 also adopt a *gauche-* conformation and the sulfate ion is stabilized by additional interactions with residues R314 and R317. The HK853 CA domain is shown in semi-transparent cartoon.

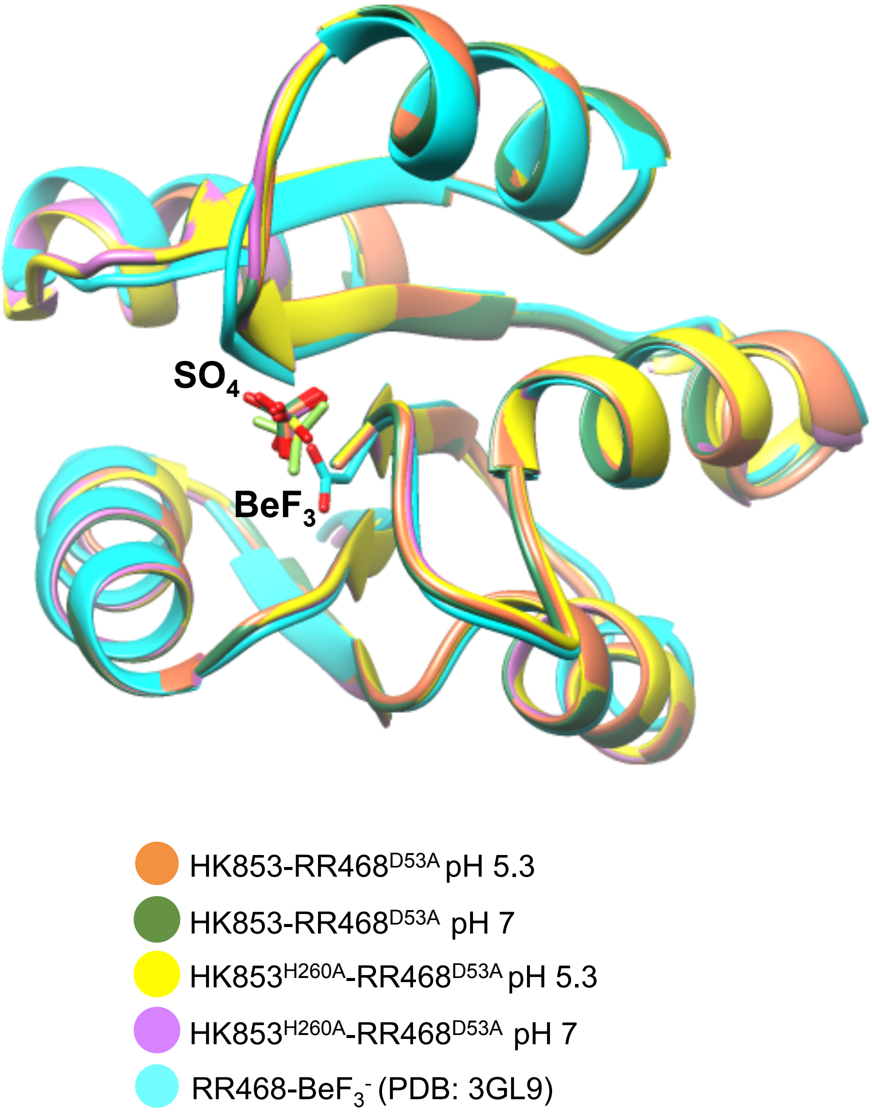

Supplementary Figure 2. A sulphate ion occupies the active center in RR468^{D53A}. The structures of RR468^{D53A} in complex with HK853 (orange and green) and HK853^{H260A} (yellow and purple) at different pHs are superimposed with the structure of the phosphorylate RR468 bound to the phosphomimetic BeF$_3^-$ (cyan, PDB 3GL9). A sulfate ion represented in sticks with the sulphur atom in the same color that the corresponding structure is found in all the structures at the same position that the phosphomimetic BeF$_3^-$ (lime).

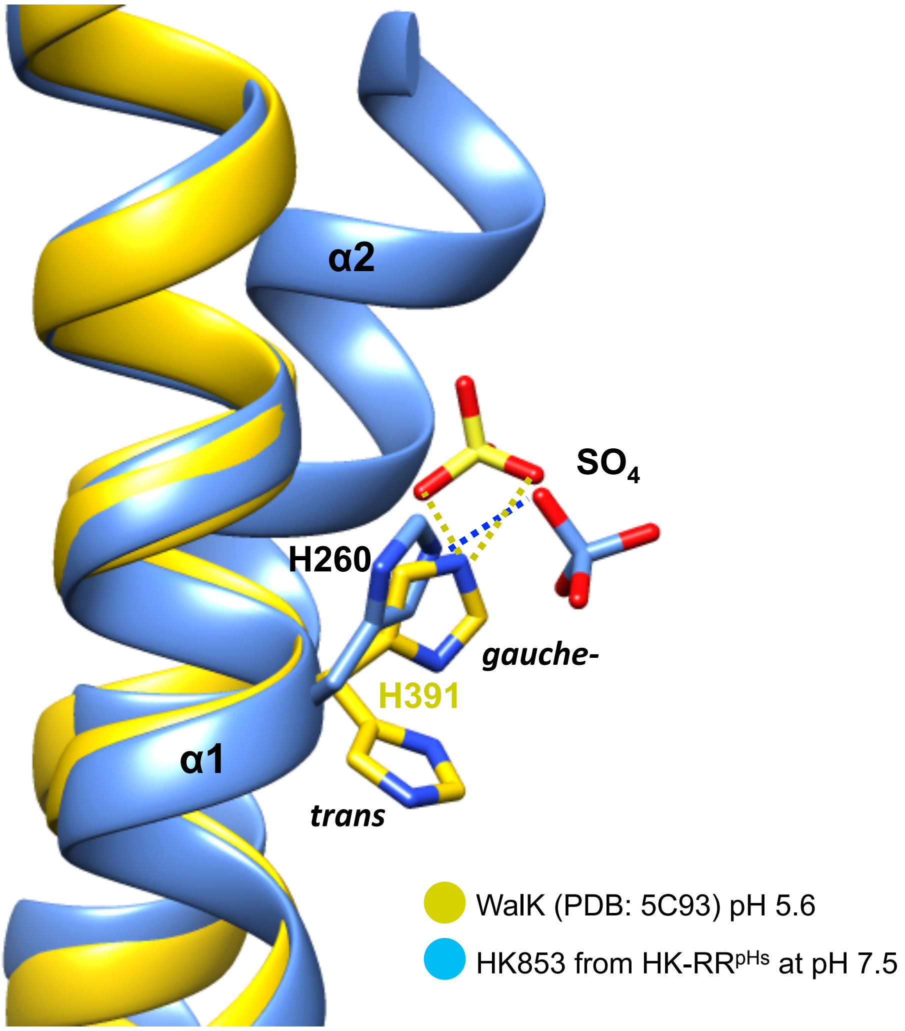

Supplementary Figure 3. H260 adopts a *gauche-* conformation to coordinate the sulfate ion. Close view of the phosphorylatable His in the superposed structures of HK853 (blue) from the HK-RR^{pHs} complex at pH 7.5 and WalK (yellow, PDB: 5C93). The DHp helices are shown in cartoon and the phosphorylatable His and the sulfates in sticks. The carbon and sulphur atoms are colored as the corresponding molecules, while nitrogen and oxygen atoms are colored marine blue and red, respectively. Interactions between the phosphorylatable His and the sulfates are denoted by dashed lines colored as the corresponding structure. Notice that Walk H391 adopt *trans* and *gauche-* conformation but only the *gauche-* conformation interacts with the sulfate.

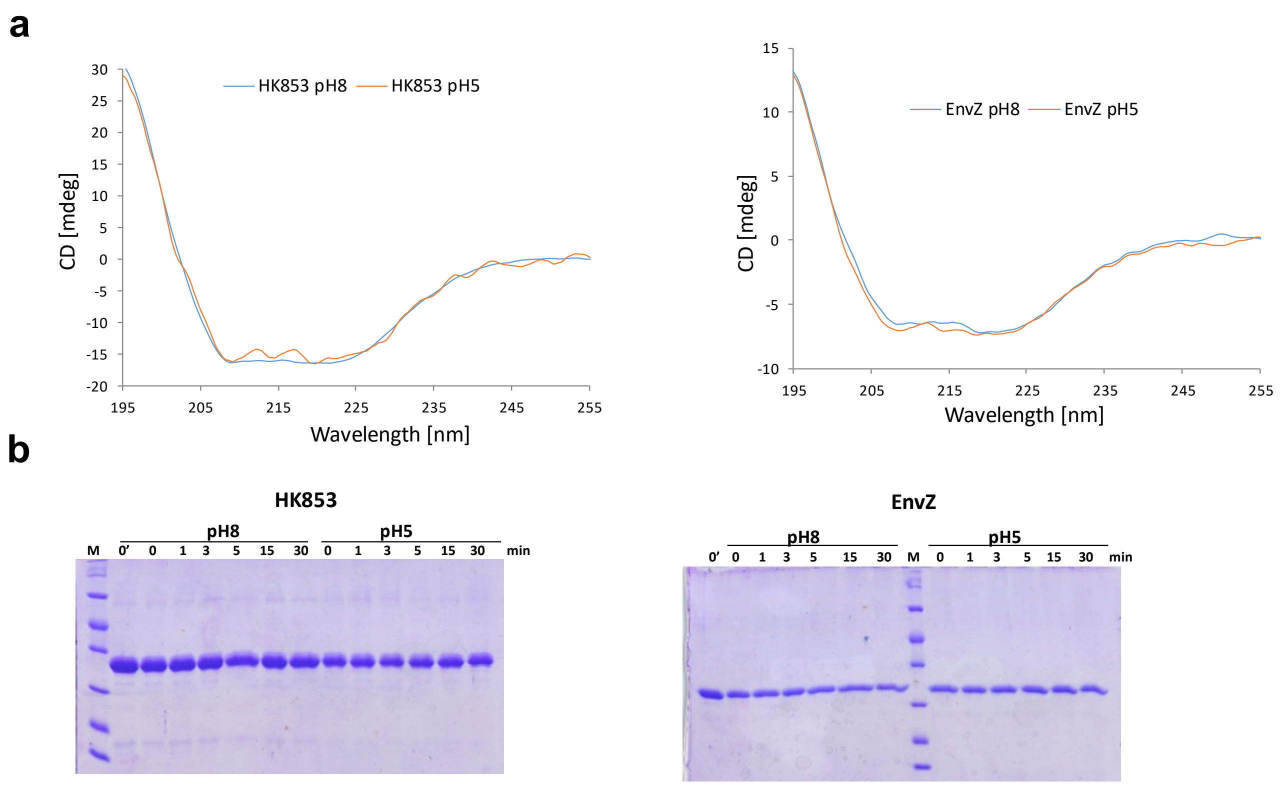

Supplementary Figure 4. Stability of HK853 and EnvZ at pH 8 and pH 5. a Circular dichroism spectra for HK853 and EnvZ at pH 8 and pH 5. The experiments were conducted in a J-1500 spectrometer with quartz cuvettes and a light path length of 0.1mm using 30 µL of protein at 0.3 mg ml^{-1}. Data was analysed with Spectra ManagerTM Suite (JASCO Corporation). **b** Coomassie staining SDS-gel showing the amount of protein loaded along the autophosphorylation stability experiment for HK853 and EnvZ at pH 8 and pH 5. M line denotes protein marker. Source data are provided as a Source Data file.

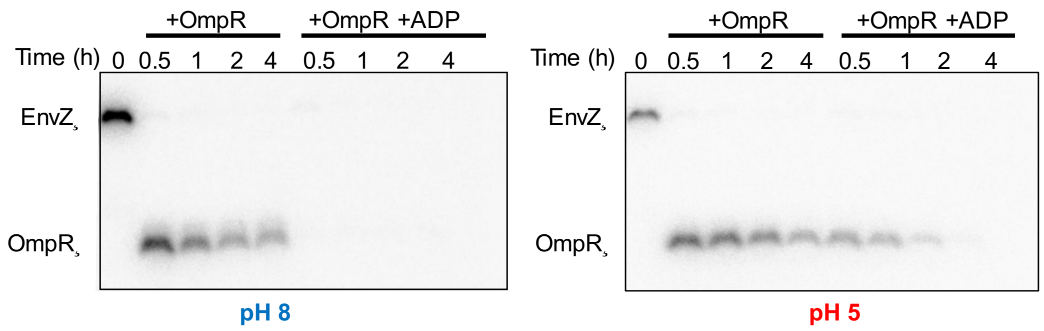

Supplementary Figure 5. Phosphotransfer of EnvZ to OmpR-REC in the absence and presence of ADP. Time course ranging from 0.5 to 4 h. of reaction at acidic (5) and basic (8) pH. Source data are provided as a Source Data file.

Supplementary Table 1. RMSD values (Å) for the superposition of the structures for the HK853-RR468 complexes.

HK853-RR468					
pH	7.0	6.5	5.5	5.0 (PDB 5UHT)	5.6 (PDB 3DGE)
7.5	0.48 (707 residues)	0.34 (708 residues)	0.34 (707 residues)	2.06 (703 residues)	0.71 (701 residues)
7.0		0.39 (707 residues)	0.44 (706 residues)	1.82 (700 residues)	0.71 (707 residues)
6.5			0.34 (706 residues)	1.92 (705 residues)	0.69 (710 residues)
5.5				1.95 (694 residues)	0.69 (710 residues)
5.0 (PDB 5UHT)					2.0 (705 residues)

Supplementary Table 2. pKa of phosphorylatable His in the corresponding structures calculated with the program ROSIE server http://rosie.rosettacommons.org/.

**Protein (PDB code)**	**Rotamers***	**pKa**
HK853-RR468 complex (5UHT)	*gauche-/gauche-*	5,9/6,0
HK853-RR468 complex (3DGE)	*trans/trans*	6,1/6,1
HK853 (2C2A)	*gauche-*	6,1
EnvZ^{Chim} (4KP4)	*trans/trans*	6,0/6,7
EnvZ ^{DHp} (5B1N)	*trans*	6,5
VicK (4I5S)	*trans/gauche-*	5,8/6,1
WalK (5C93)	*gauche-&trans/trans*	6,6/6,6
CpxA (4BIW)	*trans/gauche-*	4,8/6,1
CpxA (4BIX)	*gauche-/gauche-*	6,3/6,6
CpxA (4BIU)	*trans/trans*	5,5/5,6
HK853-RR468 complex pH 7,5(6RGY)	*gauche-/gauche-*	6,2/6,3
HK853-RR468 complex pH 7(6RFV)	*gauche-/gauche-*	6,0/6,1
HK853-RR468 complex pH 6,5(6RGZ)	*gauche-/gauche-*	6,2/6,2
HK853-RR468 complex pH 5,5(6RH0)	*gauche-/gauche-*	6,4/6,2
HK853-RR468^{D53A} complex pH 7(6RH1)	*gauche-/gauche-*	6,0/6,1
HK853-RR468 ^{D53A} complex pH 5,3(6RH2)	*gauche-/gauche-*	5,9/5,8

** Rotamer observed in the structure for the phosphorytable His.*

Supplementary Table 3. Plasmids used in this study.

Plasmid	Protein	Description
pHistag-HK853	His-HK853	HK853 catalytic domain (residues 232-489) cloned in vector pLIC-SGC1 with N-term Histag[1].
pHistag-HK853 ^{H260A}	His-HK853 ^{H260A}	HK853 complete cytoplasmatic portion with mutation H260A cloned in vector pLIC-SGC1 with N-term HisTag[1].
pHistag-RR468	His-RR468	Full-length RR468 cloned in vector pNIC28-Bsa4 with N-term Histag[1].
pRR468	RR468	Full-length RR468 cloned in vector pET22b without tag[2].
pRR468 ^{D53A}	RR468 ^{D53A}	Full-length RR468 with mutation D53A cloned in vector pET22b without tag. This work
pHistag-EnvZ	His-EnvZ	EnvZ catalytic domain (residues 224-450) cloned in vector pLIC-SGC1 with N-term Histag[1].
pHistag-OmpR $_{REC}$	His-OmpR $_{REC}$	OmpR REC domain (residues 1-122) cloned in vector pNIC28-Bsa4 with N-term HisTag[1].

References

1 Casino, P., Miguel-Romero, L. & Marina, A. Visualizing autophosphorylation in histidine kinases. *Nat Commun* **5**, 3258, doi:10.1038/ncomms4258 (2014).

2 Casino, P., Fernandez-Alvarez, A., Alfonso, C., Rivas, G. & Marina, A. Identification of a novel two component system in Thermotoga maritima. Complex stoichiometry and crystallization. *Biochim Biophys Acta* **1774**, 603-609, doi:10.1016/j.bbapap.2007.02.005 (2007).
