# Supplementary material for: Revisiting the pH-gated conformational switch on the activities of HisKA-family histidine kinases
Source: Nat Commun. 2020 Feb 7;11:769. doi: 10.1038/s41467-020-14540-5 (PMC7005713; doi:10.1038/s41467-020-14540-5)
Supplement: Supplementary file 3 — Reporting Summary [file 41467_2020_14540_MOESM3_ESM.pdf]

nature research

Corresponding author(s): Patricia Casino and Alberto Marina

Last updated by author(s): Jan 10, 2020

Reporting Summary

Nature Research wishes to improve the reproducibility of the work that we publish. This form provides structure for consistency and transparency in reporting. For further information on Nature Research policies, see Authors & Referees and the Editorial Policy Checklist .

Statistics

For all statistical analyses, confirm that the following items are present in the figure legend, table legend, main text, or Methods section.

n/a	Confirmed	
☐	☒	The exact sample size (n) for each experimental group/condition, given as a discrete number and unit of measurement
☐	☒	A statement on whether measurements were taken from distinct samples or whether the same sample was measured repeatedly
☒	☐	The statistical test(s) used AND whether they are one- or two-sided<br>*Only common tests should be described solely by name; describe more complex techniques in the Methods section.*
☒	☐	A description of all covariates tested
☒	☐	A description of any assumptions or corrections, such as tests of normality and adjustment for multiple comparisons
☒	☐	A full description of the statistical parameters including central tendency (e.g. means) or other basic estimates (e.g. regression coefficient) AND variation (e.g. standard deviation) or associated estimates of uncertainty (e.g. confidence intervals)
☒	☐	For null hypothesis testing, the test statistic (e.g. F, t, r) with confidence intervals, effect sizes, degrees of freedom and P value noted<br>*Give P values as exact values whenever suitable.*
☒	☐	For Bayesian analysis, information on the choice of priors and Markov chain Monte Carlo settings
☒	☐	For hierarchical and complex designs, identification of the appropriate level for tests and full reporting of outcomes
☒	☐	Estimates of effect sizes (e.g. Cohen's d, Pearson's r), indicating how they were calculated

Our web collection on statistics for biologists contains articles on many of the points above.

Software and code

Policy information about availability of computer code

Data collection	Synchroton Alba (Cerdanyola del Vallès, Spain) and Diamond Light source (Didcot, UK)
Data analysis	Crystallographic processing and analysis was carried out with programs from CCP4 and USCF Chimera, free and open source suites (http://www.ccp4.ac.uk/ ; https://www.cgl.ucsf.edu/chimera/). All this information has been included in the manuscript. CCP4 suite and UCSF Chimera.<br>Statistical Analyses were carried out with the scientific software GraphPad Prism. Open source software (https://www.graphpad.com/scientific-software/prism/).<br>Circular dichroism data analysis was carried out with Spectra ManagerTm Suite (Jasco Corporation; https://jascoinc.com/products/spectroscopy/spectroscopy-software/).

For manuscripts utilizing custom algorithms or software that are central to the research but not yet described in published literature, software must be made available to editors/reviewers. We strongly encourage code deposition in a community repository (e.g. GitHub). See the Nature Research guidelines for submitting code & software for further information.

Data

Policy information about availability of data

All manuscripts must include a data availability statement. This statement should provide the following information, where applicable:

- Accession codes, unique identifiers, or web links for publicly available datasets
- A list of figures that have associated raw data
- A description of any restrictions on data availability

The X-ray crystallographic coordinates reported for all the structures have been deposited at the Protein Data Bank. For HK-RRpHs, PDB accession codes are 6RGY for pH 7.5, 6RFV for pH 7, 6RGZ for pH 6.5 and 6RH0 for pH 5.5. For the mutant HK853-RR468D53A, PDB accession codes are 6RH1 for pH 7 and 6RH2 for pH 5.3 while for the mutant HK853H260A-RR468D53A, PDB numbers are 6RH7 for pH 7.5 and 6RH8 for 5.3.

Field-specific reporting

Please select the one below that is the best fit for your research. If you are not sure, read the appropriate sections before making your selection.

☒ Life sciences ☐ Behavioural & social sciences ☐ Ecological, evolutionary & environmental sciences

For a reference copy of the document with all sections, see nature.com/documents/nr-reporting-summary-flat.pdf

Life sciences study design

All studies must disclose on these points even when the disclosure is negative.

Sample size	Samples for the phosphorylation experiments were taken at different incubation times to check the increase or decrease of the intensity signal for the autoradiography.
Data exclusions	There was no need to exclude phosphorylation data
Replication	Phosphorylation experiments were performed with [γ-32P] ATP and [γ-32P] AcetylPhosphate, and were repeated at least twice in order to verify same result on each independant assay. All assays were reproducible.
Randomization	Not relevant for our study. Samples not allocated in sample groups.
Blinding	All experiments were performed without any predetermination of the result. Variable incubation times allowed us to assess clearly how the phosphorylation events were produced

Reporting for specific materials, systems and methods

We require information from authors about some types of materials, experimental systems and methods used in many studies. Here, indicate whether each material, system or method listed is relevant to your study. If you are not sure if a list item applies to your research, read the appropriate section before selecting a response.

Materials & experimental systems

n/a	Involved in the study	
☒	☐	Antibodies
☒	☐	Eukaryotic cell lines
☒	☐	Palaeontology
☐	☒	Animals and other organisms
☒	☐	Human research participants
☒	☐	Clinical data

Methods

n/a	Involved in the study	
☒	☐	ChIP-seq
☒	☐	Flow cytometry
☒	☐	MRI-based neuroimaging

Animals and other organisms

Policy information about studies involving animals; ARRIVE guidelines recommended for reporting animal research

Laboratory animals	No animals were used in this study
Wild animals	No animals were used in this study
Field-collected samples	No field-collected samples were used in this study
Ethics oversight	No ethical approval or guidance was required

Note that full information on the approval of the study protocol must also be provided in the manuscript.
